# A Review of Characterization Approaches for Smallholder Farmers: Towards Predictive Farm Typologies

**DOI:** 10.1155/2019/6121467

**Published:** 2019-05-22

**Authors:** Devotha G. Nyambo, Edith T. Luhanga, Zaipuna Q. Yonah

**Affiliations:** Information and Communication Science and Engineering, Mandela African Institution of Science and Technology, P. O. Box 447, Arusha, Tanzania

## Abstract

Characterization of smallholder farmers has been conducted in various researches by using machine learning algorithms, participatory and expert-based methods. All approaches used end up with the development of some subgroups known as farm typologies. The main purpose of this paper is to highlight the main approaches used to characterize smallholder farmers, presenting the pros and cons of the approaches. By understanding the nature and key advantages of the reviewed approaches, the paper recommends a hybrid approach towards having predictive farm typologies. Search of relevant research articles published between 2007 and 2018 was done on ScienceDirect and Google Scholar. By using a generated search query, 20 research articles related to characterization of smallholder farmers were retained. Cluster-based algorithms appeared to be the mostly used in characterizing smallholder farmers. However, being highly unpredictable and inconsistent, use of clustering methods calls in for a discussion on how well the developed farm typologies can be used to predict future trends of the farmers. A thorough discussion is presented and recommends use of supervised models to validate unsupervised models. In order to achieve predictive farm typologies, three stages in characterization are recommended as tested in smallholder dairy farmers datasets: (a) develop farm types from a comparative analysis of more than two unsupervised learning algorithms by using training models, (b) assess the training models' robustness in predicting farm types for a testing dataset, and (c) assess the predictive power of the developed farm types from each algorithm by predicting the trend of several response variables.

## 1. Introduction

The exact definition of what a smallholder farmer means varies widely worldwide depending on location and intensification of farming systems. Generally, a smallholder farmer is viewed as a person involved in farming a small piece of land, cultivating food crops, sometimes with small varieties of cash crops [1–3]. In many localities, smallholder farmers practice mixed crop-livestock farming, whereby the number of large ruminants kept is around 3-5 [2]. Operations in such farms are at large managed by family labor, since the primary intentions for farming are dedicated to support internal family needs. Smallholder farmers (owning less than 2 ha of land) constitute the largest proportion of the 570 million farms worldwide [4]. In Africa, they dominate the agricultural sector and contribute about 75% of agriculture production and 50% of the livestock products. Despite the presence of abundant land in places like Africa, ownership of land by smallholder farmers has been decreasing in size and is expected to continue decreasing in the long run [5, 6].

Characterization of smallholder farming systems refers to describing the various categories of farms—their demographics, attributes, production trends, and existing production systems. Through characterization, existing farming systems within a study case can be studied. Generally, characterization of farms/farming systems involves determination of classes of farms/farming systems, whereby each class exhibits different attributes. In other terms, classes obtained in a characterization process are known as production clusters. The main goal of characterization is to depict production categories existing in a particular environment or a complex agroecological system for appropriate introduction of improved technologies and conversant policy support [7]. Mostly, in advanced analysis of farms/farming practices, development of typologies is crucial to avoid aggregation. The subgroups unveil existing variations among farmers or farm types and therefore an improvement plan can be targeted to a particular group of farms/farmers instead of aggregating them as one.

Various methods are reported in the literature regarding smallholder farms/farmers characterization, all of them ending up with development of some subgroups from a dataset. Methods range from participatory to advanced statistical and machine learning algorithms with increasing order of complexity. The algorithms used in characterization can be grouped into deterministic approaches or probabilistic approaches. In deterministic approaches, given the same seeding and fixed number of classes, an algorithm will produce the same type of results. On the other hand, probabilistic approaches will change the grouping of members even without changing the seeds or the number of clusters. Mostly, the deterministic approaches use a kind of supervised learning, while the probabilistic approaches use unsupervised learning. In unsupervised learning, an algorithm would self-group the dataset based on specific conditions for that algorithm.

Smallholder farms/farmers have extensively been characterized using various approaches and some approaches are being overly used over the years, while others remain unexplored. Different areas of application for deterministic and probabilistic methods in characterization are presented to highlight factors that should be considered in selection of an approach to group farmers. Advantages and disadvantages of supervised and unsupervised algorithms in characterization are presented, leading to a hybrid approach being recommended to characterize smallholder farms and develop predictive farm typologies.

## 2. Methodology 

Analysis of related literature in characterization of smallholder farmers comprised of three main steps. The first step involved establishing a general understanding on characterization of smallholder farmers and methods used. Various approaches were observed from summary review of retrieved abstracts. The approaches were categorized as either deterministic or probabilistic approaches whereby the deterministic approaches involved more of supervised algorithms, while the probabilistic approaches involved more of unsupervised algorithms. In the second step, keywords were defined to enable a search of related works involving the use of the following methods: regression analysis, chi-square test, discriminant analysis, combinatorial method, expert-based and participatory characterization, hierarchical and nonhierarchical cluster analysis, naïve Bayes clustering, and fuzzy and Self-Organizing Maps (SOM) cluster analysis. Search query used was “*characterization of smallholder AND regression analysis OR chi-square OR discriminant analysis OR combinatorial method OR expert based characterization OR participatory characterization OR hierarchical clustering and non-hierarchical clustering OR naïve Bayes clustering OR fuzzy clustering OR Self-Organizing Maps.” *The search was done for archives in ScienceDirect and Google scholar for articles published in 2007 - 2018. The initial number of returned articles from the searches was 87. Through review of abstracts, articles indicating formulation of farm typologies from characterization were the ones retained for detailed review, and 20 articles were retained.

## 3. Overview of Smallholder Farmers Characterization 

Smallholder farmers have been extensively characterized with the intension of finding out farm typologies and characteristics of each typology to inform decision making regarding improvement of farming systems. For most of the reviewed literature, factors considered in smallholder farms characterization include, but are not limited to demographic data, labor availability and work distribution, herd structure, facilities and machinery, feeding management, reproductive and milking management, health management, supply of inputs, area under cultivation, usage of fertilizer, and usage of concentrates. Four main groups of methods have been widely applied to characterize smallholder farms. These are deterministic, probabilistic, participatory, and expert-based methods. However, algorithms used in deterministic and probabilistic methods vary widely as well and lead to some uniqueness of each application. A review of the literature on the differences between usage of deterministic and probabilistic methods shows that most applications of probabilistic approaches, such as clustering, remain to have an academic advantage rather than advantages on business processes [8]. The main cause of this difference is the unpredictability of the methods; that is, two methods would yield different characterization results.

### 3.1. Deterministic and Probabilistic Methods

Discriminant analysis, combinatorial method, and logistic regression are among the commonly used deterministic algorithms to characterize smallholder farmers. Hierarchical and nonhierarchical clustering are the most common clustering algorithms based on probabilities. In addition to these, self-organizing Kohonen algorithm, naïve Bayesian, and fuzzy classification are also reported in the literature as best approaches to characterize farming systems. However, popularity of the latter set of algorithms cannot be compared with the first (common) ones due to the complexity involved. The packages for these algorithms are included in most of the open source and commercial statistical software. In most applications of probabilistic characterization, these algorithms are unsupervised, although some may also be applied in a supervised manner [9, 10].

### 3.2. Participatory Methods

Participatory approaches in farms classification involve the knowledge of the farmers in specific localities to describe and categorize their farming systems based on their experiences and social-cultural values. Inclusion of indigenous knowledge in the characterization process reveals some patterns that could be hard to explain or reveal statistically [11]. For example, differences in cultural values on how women are related to household activities, including agricultural, could be different among study cases. However, through statistical characterizations, these facts cannot be captured or their explanations can be weak [11]. Therefore, it is acknowledged that scientific knowledge generated from statistical tools can be interpreted based on local knowledge [12].

### 3.3. Expert-Based Methods

These methods involve use of domain experts to validate or define what is expected to be a correct classification of farms in a region. Biarnès, Bailly & Boissieux [13] studied through expert-based tree partitioning method the variations of agricultural practices in vine farms. Van de Steeg et al. [14] characterized land use and farming systems and made use of experts to validate the classification made. Use of experts in formulating farm typologies is tedious depending on the number of datasets available. For that reason, expert-based methods have been recommended mostly to be used to validate classifications done by expert systems/algorithms [14].

## 4. Deterministic Methods for Smallholder Farmers' Characterization

Spatial variation in environment and socioeconomic conditions has also been used to characterize farming systems to avoid the need of mapping all farming systems in a big region. The study done by [14] used a classification algorithm and regression models to characterize farming systems from Kenya highlands. Parameters used for classification were area under cultivation of food and cash crops, milk production, and usage of fertilizers. Classes of farms were formed and their variability was explained by location factors and household characteristics, by fitting them in a logit model. The authors' consideration for spatial parameters was influenced by gaps produced in other studies on land use and land cover changes which did not introduce spatial factors in their models. Although some studies detailing use of spatial data, land cover, and human population existed, these parameters could not fit for a more localized and specific characterization such as the smallholder dairy farm. After having variable farming systems, given location and household characteristics, the likelihood of finding a particular farming system in the area was estimated. Expert-based classification was used to create different farming systems, as a method to validate the estimates done by the statistical method. A confusion matrix was used to compare the field validation data against the estimates done by the statistical model.

The use of Principal Component Analysis (PCA) has been reported by Riveiro-Valiño et al. [15] for variables selection before use of Combinatorial Methodology (CM) for farms classification. The combinatorial method was used to classify farms' data collected in agriculture census in Galicia, Spain. The study proved use of combinatorial method as a suitable method for classification models by comparing results obtained from Discriminant Analysis (DA) method for the same datasets. Classes obtained by using CM and DA were the same. Use of DA is also supported by Gizaw et al. [16], in which farming systems developed based on demographic data were validated. Validated classification of smallholder dairy farmers placed farmers into three categories (urban, rural, and periurban systems). According to Gizaw et al. [16], gaps left by the demographic based classification includes valid statistical analyses to support the classification, missing farm topologies in the different systems and within system homogeneity to support aggregation. Apart from characterization, understanding determinants of variation in herd structure within systems was an important aspect in the Gizaw et al. [16] work. A linear combination of four predictor variables was considered, including local, low, and high grade cross-bred cattle. A discriminant function based on all the four variables was used to validate classification of the farms into the three categories. Gizaw et al. [16] report a significant proportion of misclassified samples (periurban systems) by using discriminant analysis. However, correct classification of other samples was in line with the original classification based on demographics data in urban and rural systems. Results from the classification done by Gizaw et al. [16] are showing a need to validate classes by employing diverse methods, such as clustering.

Berkhout et al. [17] used regression analysis together with a bioeconomic model to characterize various farmers in terms of heterogeneous priorities and emphasis on sustainable land use. Despite the fact that farmers aim at increasing their utility values, the heterogeneity in their preferences and production goals should not be overlooked [18]. As a method of characterization, regression analysis determines the existing relationships among variables and how the variables affect or influence each other. Through the characterization, farmers whose soil had favorable nutrients balance are those who were market oriented and put more emphasis on sustainable land use. Heterogeneity in goals of production had a direct linkage to available soil nutrients and sustainable land use practices. Berkhout et al. [17] described and used a utility function for individual households (which is related to the households' production goals). The Multiattribute Utility Theory (MAUT) was used to estimate the households' utility from cropping a particular pattern. Regression analysis was specifically used to evaluate the influence of variables used in utility estimation. The used approach is very useful when characterizing farmers based on highly correlated variables and therefore makes regression analysis a feasible method of determining the variables' relationships.

## 5. Probabilistic Methods for Smallholder Farmers' Characterization

Mburu et al. [19] carried out a study to characterize smallholder farmers in the highlands of Kenya for livestock improvement. Smallholder dairy farms were classified in terms of risk management strategies, household resources, dairy intensification, and access to services and markets. The main goal for clustering was to understand the constraints and opportunities within smallholder dairy farming systems. Principal Components Analysis (PCA) together with the cluster analysis was used to generate four categories of farmers in the study area. PCA is a statistical method for data reduction without removal of important information. All variables used in the PCA and cluster analysis were grouped into level of intensification of the dairy system, risk management strategies, level of access to output markets and input services, and household resources. The four themes had principal components that were analyzed and compared across the formed clusters. Validation of farm clusters is deemed important especially when developed from use of unsupervised algorithms as explained by Kuivanen et al. [20].

Generally, cultural values can influence classification of farming systems based on locations. Use of statistical methods alone for clustering may limit their effectiveness to represent the local reality and result in some unrealistic groups. A study by Kuivanen et al. [11] introduced a combination of statistical and participatory approaches to classify farming systems in Northern Ghana. Six topologies were developed from use of a statistical method, while five were developed from use of a participatory method. A major cause of the difference in the number of topologies developed was that the statistical method assumed the entire household as a single unit, while the qualitative participatory method assumed several types of farmers per household. Variables selected for the statistical method were from household characteristics, labor, land use, livestock ownership, and income dimensions. Cluster analysis was done to have the PCA divided into several k-groups. Farmer groups obtained from the cluster analysis were determined by a hierarchical agglomerative algorithm and were refined by a nonhierarchical partitioning algorithm. Farmers' involvement in the participatory method resulted in categorization of the farmer groups with regard to cultural values, which could not be identified/considered while using the statistical method. The use of participatory method can be seen as a method to validate the probabilistic clustering method. Kuivanen et al. [11] recommend use of both methods for farmers' classification as the methods complement and validate each other.

On the other hand, probabilistic characterization methods have been used to validate expert-based classification of smallholder farms. Dossa et al. [21] characterized urban and periurban smallholder farmers to validate an expert-based classification of the same farmers (that was based on farm assets) by using PCA and clustering methods. The authors considered the pitfalls of linear relationships assumption of the standard PCA algorithm for features selection. The results from PCA analysis determine clustering outcomes, so the features selection process (for a highly dynamic dataset) should consider linear and nonlinear relationships among data variables. Categorical PCA (CATPCA) was used for features selection to overcome the pitfalls of the standard PCA algorithm. Dossa et al. [21] also presented use of a nonhierarchical two-step clustering algorithm which works differently from the classical Ward method. A comparison of the two clustering algorithms suggests that the nonhierarchical two-step algorithm allows use of categorical (must be multinormally distributed) and continuous variables (must be normally distributed). While the classical Ward method does not provide optimal number of clusters as suggestions, the two-step nonhierarchical method does provide an optimum number of clusters to be formed.

Hierarchical Ward's and partitioning methods of Cluster Analysis (CA) have also been reported by Bidogeza et al. [18] as good approaches to verify on optimal number of clusters. Bidogeza et al. [18] used a mixture of categorical and quantitative variables to characterize and form farm topologies in Rwanda. The main goal of the study was to understand farming practices of various farmer topologies in the study area. Education, risk perception and attitude, farm size, labor availability, and land ownership are among the variables which were considered for characterization by using PCA and CA algorithms. The categorical variables used in PCA were converted to dummy variables (binary data types) and analyzed together with the quantitative variables. Factors used in CA were constructed through PCA and orthogonally rotated in order to load a small number of correlated variables into the factors. Retained variables were in accordance with the Kaiser criterion (eigenvalues higher than one). As a method of validating the CA process, the dendrogram from Ward's method was used for expert-based validation on the formation of the farm topologies. It is important that the variables causing great variability among clusters be identified [22]. Bidogeza et al. [18] used the Levene test (one-way analysis of variance) to identify the variables causing great variability among clusters. The same methods for variables reduction and clustering are described by Goswami et al. [7]. In addition to work by Bidogeza et al. [18], Goswami et al. [7] used two steps in clustering. In addition to the Ward method, the K-means algorithm was used to fine tune clusters.

The hierarchical Ward method is also explained by [23]. The authors detail that heterogeneity in smallholder dairy systems can be in various aspects including socioeconomic and farm characteristics, farmer's wealth status, availability of extension services, available sources of information, and the importance of a particular technology to the farmers. Galdino et al. [23] characterized smallholder farms in central Mexico based on farm and socioeconomic characteristics by using the hierarchical Ward method of clustering. Variables used for the CA were reduced by the factor analysis method of PCA. Validation of the clusters is not explained in the paper. In addition, Galdino et al. [23] acknowledged various clusters formulated in other researches for the same study area. The variability in the number of clusters formed in various researches proves the fact that probabilistic approaches in characterization will always yield different results even for a slight change in the number or type of variables used [8]. Although Ward's method has some drawbacks especially on the structure of dataset and presence of missing values, it has been reported to be the best method in a recent study to characterize dryland farming systems [24] whereby missing values were estimated by using nearby values.

Bernués & Herrero [25] work on characterizing farming systems in Bolivia likewise involved use of CA to formulate farming systems. However, a different approach for variables reduction was used: the Multiple Correspondence Analysis (MCA). The goal of MCA is the same as the PCA, to reduce variables, while maintaining important information where noncorrelated variables are considered to explain classes' variability. Bernués & Herrero [25] also used the hierarchical approach for clustering based on centroids Euclidean distances whereby the number of clusters is validated by a strong increment in the cubic criterion for clustering (CCC) and pseudo-F values and a strong decrement in the pseudo-T value. Main differences between Bernués & Herrero [25] and Bidogeza et al., [18] are in the clusters validation approaches; while Bidogeza et al.'s [18] approach to retain factors is based on the eigenvalues, Bernués & Herrero's [25] approach is not explained.

Kuivanen et al. [20] used PCA, hierarchical Ward, and nonhierarchical partitioning methods to characterize smallholder farmers/types in Ghana. The method for PCA presented by Kuivanen et al. [20] detailed the effect of missing values and outliers in a dataset and so they were removed before the PCA procedure. As a result of that truncation of data, about 12.5% of the dataset was lost. However, as a prerequisite to PCA and CA, missing values and outliers must be removed. From the PCA results, factors considered in selection of the principal components in addition to Kaiser's criterion for eigenvalue were (a) minimum cumulative percent of variation (60% was chosen) and (b) correlation between variables and the principal components (highly correlated variables were considered for interpretation, >0.50). The number of groupings was defined by the Ward method, while a nonhierarchical partitioning method was used to refine the number of groups. Kuivanen et al. [20] also elaborated the use of farm experts to validate groupings obtained from the CA.

Classification and characterization procedures that limit a household or farm to belong into only one cluster are termed as hard by Salasya & Stoorvogel [26]. Salasya & Stoorvogel tested for the first time the use of fuzzy classification on farming households. The key difference between fuzzy classification and other techniques for classification is that a member may belong to more than one cluster or not belong to any cluster at all. A probabilistic membership value produced in the fuzzy process is used to explain belongingness of a member into a particular cluster. Comparing fuzzy classification with other common approaches for characterization, it is difficult to tell how well a member fits into a particular cluster since it is strictly demanded by the methods that a member must belong into one cluster [26]. Therefore, use of a membership value correctly determines how well the member is linked to a particular cluster. The approach is particularly important if membership in a cluster can be altered by a minor change in one of the variables involved. Fuzzy K-means was used in the implementation whereby within-class sum of square error is minimal. Degree of fuzziness is given by the fuzzy exponent whereby it explains how fuzzy is the solution produced. Ranging from 1 to ∞, the value of 1 indicates belongingness into only one cluster. As the degree increases to ∞, classification of such a member becomes highly fuzzy. This is unlike the Boolean logic given to the common clustering “true or false.” The membership value is inversely proportional to the degree of fuzziness. Based on the results, Salasya & Stoorvogel [26] conclude that the fuzzy classification is suitable when an intention is to find out extremes rather than averages. In addition to work done by Salasya & Stoorvogel [26], fuzzy classification can be applied to classify qualitative datasets by using the fuzzy C-means algorithm [27].

Specifically, use of fuzzy classification in farming systems and activities has been on soil classification, crop suitability, and weather-related studies [28–30] (Didier et al., 2010).

While Salasya & Stoorvogel used quantitative datasets, Pelcat et al. [31] used fuzzy clustering for satellite images to find cost effective means of allocating management zones to fields. This kind of characterization is beneficial to farmers as it results in effectiveness of cropping inputs. Pelcat et al. [31] proved that satellite imagery and fuzzy K-means can be combined to characterize fields. Not differing from Salasya & Stoorvogel [26] implementation, the use of membership values (determined by the Euclidean distance between a point and the centroid) and the fuzzy exponent determined the correctness of the clusters. From work done by Salasya & Stoorvogel [26] and Pelcat et al. [31], fuzzy clustering can be implemented with a small number of datasets.

Smallholder farms' classification into fuzzy class boundaries is also supported by Paas & Groot [32] in which Naïve Bayesian (NB) methodology was used to classify farms types. Use of NB avoids formulation of farm types based on frequentist statistics where possibilities of a farm to belong into more than one category are not explored, and since the classification does not depend on frequencies, NB classifiers do work with a minimum number of datasets or variables [32]. While the fuzzy classification by Salasya & Stoorvogel [26] explained the importance of the membership value produced during the fuzzy process, NB uses a likelihood as a probability of an observation/member to belong into a certain farm type. The posterior likelihood is defined as

(1) $$\begin{array}{c} Posteror=\frac{Prior\times Likelihood}{Evidence} \end{array}$$

The prior, likelihood, and evidence are all derived from a training dataset in which Paas & Groot [32] derived 9 samples of the dataset as training set (10%......90%), leaving the unselected sets for NB classifier validation. It is reported that the bigger the size of the training set, the smaller the standard deviations in the classes, and misclassification was mainly observed when 10% and 20% of the dataset were used in training the NB classifier. Underrepresented farm types in a training set may be wrongly classified. For this reason, Paas & Groot [32] recommend use of a participatory approach to validate farm types and propose possible cluster numbers and prior probabilities for each cluster. Use of participatory methods to complement probabilistic clustering/classification is equally supported by Kuivanen et al. [11] and Baltenweck et al. [3].

Naïve Bayesian (NB) and Random Forest (RF) algorithms are termed as the best to classify object-based images in a supervised approach. Work done by Dimov [10] classified small-scale cropping systems mainly garden and summer plots by using fused satellite images. The NB and RF are among the used algorithms to find out whether image fusion increases classification accuracy. PCA was used to orthogonally transform variance of all the input bands whereby the first component contained most of the variance. The eigenvalue criterion was used for the components, in which the remaining components contained uncorrelated information, reduced variance and also they decreased in common variance. The authors commended the NB method for its ability to classify based on few parameters and a reduced complexity. Like Paas & Groot [32], Gaussian distribution is assumed.

Bayesian classification algorithms have been used widely in soil classification and crop yield assessment [33–36].

Dryland farming systems have been studied and characterized through clustering by Nazari et al. [24] by comparing effectiveness of three clustering methods (hierarchical Ward's, nonhierarchical K-means, and Self Organizing Maps (SOM)-Kohonen). Quantitative weather data were used in the study and in the presence of missing data from some weather stations. Estimations were done using data from nearby stations. In the reported study, estimations of cluster numbers from the SOM method were achieved through dendrograms from the Ward methods; this is commended as the best way to naturally detect cluster numbers [24]. However, validation of the results must be done by comparing outputs from other methods.

In work done by Nazari et al. [24], the SOM-Kohonen as an approach based on Neural Networks (NN) was used to form dryland farming systems' clusters. Basic principles of the Kohonen algorithm [37] are presented by Gelbard et al. [8]. For a given dataset, the algorithm will generate a two-dimensional NN whereby each neuron is connected to each record and other neurons surrounding it. The algorithm assigns weights to the records and neurons; when records are passed through, weights are compared and a neuron most similar to the passed record (Best Matching Unit, BMU) wins the record. Neuron weights are updated every time a neuron wins a new record. The use of NN to characterize farming systems is not as popular as NN applications to hydrology and water resources [24]. The implementation of the SOM can be a combination of SOM-K-means, SOM-average, or SOM-Ward as done by Nazari et al. [24]. The performance of the SOM in comparison with the other algorithms was best when it was combined with the Ward's and the average method (which considers the distance between pairs of records in two pair of clusters [24]). Based on the nature of the datasets (quantitative and no missing records), it is not a surprise that the Ward method performs better.

Also, the Kohonen SOM has been used in characterizing smallholder farms in Italy to establish whether farm size can determine efficiency and farm net income [38]. Datasets used in the study were quantitative and the variables were farm size, land capital, labor capital, and invested capital and subsidies. SPICE-SOM software was used for competitive training of the neurons in which a Euclidian distance of all input data and output nodes was determined [38]. Kohonen et al. [39] detailed that for any neuron to win a vector (record), it must satisfy

(2) ∀ni∈S:diffnwinnerweight,v≤diffniweight,v

whereby v is any new weight vector, n_winnerweight_ is the current weight of the winning neuron, and n_iweight_ is a weight of any other i^th^ neuron on the map. Reference [38] explains the importance of learning coefficient for the neurons as they determine change of weights for each BMU. The learning rate takes a form of a decaying function and is dependent on the distance of neurons from BMU [38]. By using the SOM, [38] characterized the efficiency and total gain from small family farms, cooperative and limited company farms. From the results, SOM indicated that there is an influence of farm size on farm efficiency and total gain.

## 6. On Farm and Expert-Based Methods for Smallholder Farms Characterization

Herrero et al. [3] support that characterizing smallholder agricultural producers and their systems is crucial in understanding the farms evolvement. Their study took a holistic approach to determine evolvement of crop-livestock farming systems by integrating macrolevel socioeconomic drivers, regional-level, land use patterns, and household dynamics to predict how the system might evolve in two decades. Expert-based classification of farmers was done by using predetermined criteria. The output from the farms classification was validated through hierarchical cluster analysis by using Statistical Analysis Software (SAS) and the same results were revealed. Household dynamics were modeled by using a linear programing algorithm aimed at maximizing the farm's gross gain. The LP model was adapted for multitime period modeling in which results from the annual optimization of the household gains were used as inputs for next year's run.

Therefore, it is acknowledged that evolvement can be determined by the presented expert-based methods. However, the segmentation of the approaches to achieve the common goal of modeling evolvement should not be overlooked. Nolan et al. [40] present a complex case in the real world in which several models must be integrated to deduce a conclusive outcome. Furthermore, evolvement of crop farming and livestock keeping need to be considered as they may influence or affect each other. Modeling such systems by using econometric approaches may result in failure to capture the real-world heterogeneous nature of smallholder farms [40].

Involvement of dairy farmers into initiatives for breed improvement programs motivated the study done by [41] that involved an on-farm characterization of production systems of selected cattle breeds. Summary and descriptive statistics were used to compare production systems of two cattle breeds in Sudan (Butana and Kenana breeds) preceding a survey done on two areas keeping the cattle breeds. Data from the survey was analyzed separately based on area to allow easy comparison of the results. Chi-square test was used to approve the significance of results. The method used by [41] provided a precise characterization and an in-depth differentiation of the husbandry practices and production constraints given that the study areas were defined by the cattle breeds in study. The method could not be applied to a sample of farms that practices mixed types of breeds and practices due to heterogeneity and dynamic nature of the farms.

## 7. Discussion

Various methods used to characterize smallholder farming systems have been studied, ranging from expert-based, participatory, deterministic, and probabilistic approaches. The literature indicates some similarities in all methods such as the use of multiple variables for characterization, features reduction by using PCA, and reliance on domain experts for farm types' validation. However, all methods used lack an important component of validating the usefulness of the farm types:* How well can the developed farm types be used to predict future trends of the farmers?* Nonetheless, the use of one method to classify and form farm types is straightforward and does not require much of statistical knowledge for validation as they rely mostly on domain expert validation. Use of domain experts assures that developed farm types reconcile with the farms in the real world. In some cases where several algorithms are compared [10, 15, 20, 24], the key target has been to observe how well they can create distinct farm types and not how robust and useful are the formulated farm types in studying the future of farming. Although the choice of a characterization method depends on the goals of an analyst, in order to understand the nature of farming systems in their generality, use of unsupervised algorithms outperforms the supervised algorithms by providing all available groups of farmers as seen in Kuivanen et al. [20] and Nazari et al. [24].

Unsupervised learning presents a probabilistic characterization of smallholder farms. Unlike the classification approaches which are more deterministic, clustering has been extensively used in academic research and data analysis that is not directly linked to commerce and business applications. Gelbard et al. [8] present some reasons for the slow adoption of unsupervised learning methods in commerce and business management. Firstly, there is lack of standards in clustering algorithms, which is faced in two dimensions: different clustering algorithms producing different results [42] and no standard of identifying appropriate number of clusters [18], but rather basing the number on averages [43]. This unpredictability and inconsistent nature of clustering methods has resulted in lack of awareness on the value brought by cluster analysis [8]. Secondly, there is no clear definition of the clusters interpretation and the implementation. Also, there is no clear information on how to select an appropriate algorithm and how to interpret related results. In addition, universal methods to compare effectiveness of the algorithms are missing [8]; some evaluation metrics are limited to particular algorithms.

Clustering algorithms are unpredictable because they use probabilities, so the nature of data, order of data, and defined number of iterations contribute to the unpredictability. Clustering methods can complement each other, and thus use of more than one method to group data is recommended for accuracy [20]. On the other hand, classification algorithms (highly deterministic) are dependent on training datasets. These algorithms which fall on supervised learning will always observe the data pattern and outcomes from training set to work on any dataset with similar attributes. This class of algorithms has been used widely in analyzing business trends and customer relationships due to their high predictive power.

Considering the advantages of both approaches, supervised learning approaches can be used to validate farm types developed from unsupervised learning. As such, characterization of farms can be banked on three steps: (a) develop farm types by a comparative analysis of more than two unsupervised learning algorithms by using training models, (b) assess the training models' robustness in predicting farm types for a new dataset, and (c) assess the predictive power of the developed farm types for each algorithm. Through this, the stability of a clustering model can be assessed and compared, and predictive power of the farm types can also be assessed. The recommended approach has been tested and the reader is referred to [44] for more details. The proof of concept has been done by comparing K-means, fuzzy clustering, and Self-Organizing Maps (SOM) algorithms to cluster and assess the predictive power of final clusters of smallholder dairy farmers.

Further research is required on how unsupervised learning models can be implemented to produce stable features suitable for evolvement studies. For example, in agricultural systems, it is highlighted in previous research that evolvement trends of such systems be established if poverty alleviation goals and food security are to be met [3]. In the proof of concept [44], the authors have reported use of three clustering algorithms that yielded different accuracies and predictive ability. It is necessary to further assess how the nature of different datasets affects performance of the tested algorithms. With an understanding on how datasets affect performance of unsupervised algorithms, further studies can be directed into protocols for suitable data collection/aggregation and preparation prior to characterization and study on the systems' evolvement trend.

## 8. Conclusion

The literature on approaches used to characterize smallholder farming systems has been presented. Commonly used approaches ranging from deterministic to probabilistic are presented with much reliance on domain expert validation of farm types. Participatory approaches relying much on local knowledge have also been observed. A key challenge in the reviewed approaches towards characterization of smallholder farming systems is lack of validation methods and metrics to prove on robustness of farm types and their effectiveness in predicting future trends of farming. Unsupervised learning approaches have shown to provide more dynamic farm types which are entirely based on nature of datasets. In view of the strengths and weaknesses of unsupervised learning, use of supervised learning approaches to validate developed farm types is deemed important to ensure stability of unsupervised models and predictive usability of developed farm types. Recommended approach assumes an objective validation process which can also help nondomain experts to interpret farm types. In view of the presented literature and referred proof-of-concept paper, the authors suggest areas for further research in order to have a standard method for characterizing farming systems based on unsupervised algorithms.
